# Selection-free endogenous tagging of cell lines by bicistronic co-expression of the surface antigen NGFR

**DOI:** 10.1016/j.mex.2022.101929

**Published:** 2022-11-18

**Authors:** Marcel Seibert, Nina Kurrle, Verena Stolp, Heike Nürnberger, Sandra Tzschentke, Leon Börner, Frank Wempe, Hubert Serve, Frank Schnütgen

**Affiliations:** a Department of Medicine, Hematology/Oncology, University Hospital Frankfurt, Goethe-University Frankfurt, Frankfurt/Main 60590, Germany; b German Cancer Consortium (DKTK), Partner Site Frankfurt/Mainz, and German Cancer Research Center (DKFZ), Heidelberg 69120, Germany; c Frankfurt Cancer Institute, Goethe-University Frankfurt, Frankfurt/Main 60596, Germany

**Keywords:** CRISPR, Endogenous protein tagging, Physiological, Protein-protein interactions, Exogenous co-expression of surface antigens

## Abstract

Endogenous protein tagging, in contrast to exogenous overexpression of tagged proteins, allows to characterize specific protein functions under defined physiological or pathophysiological conditions without the influence of non-physiological protein levels. The development of generic and homology-independent tagging strategies, exploiting the CRISPR/spCas9 gene editing system in combination with generic tag donor plasmids, allows targeted and precise gene modification in mammalian cells for almost any desirable gene.

So far, fluorescent tags or antibiotic resistance cassettes coupled to the endogenous fusion protein expression have been applied to isolate correctly modified clones. However, both can be challenging, especially when endogenously controlled expression of the tagged protein is weak or regulated by cellular signals. Here, we expand the strategy to selection-free endogenous tagging by exploiting exogenous co-expression of surface antigens. These endogenously regulated, but still easily accessible surface antigens allow simple identification and isolation of clones harboring correctly tagged alleles via common sorting procedures (e.g. FACS/MACS).

Using metabolically controlled interaction studies of the endogenously tagged mTORC1-regulating GATOR2 complex protein WDR59, we show that endogenous GFP-labeling does not affect complex association of fusion proteins and downstream signaling via mTORC1. In addition, exogenous co-expression of the NGFR surface antigen does not influence conditional protein-protein interactions.

• A method for selection-free, site-specific, homology-independent endogenous genetic tagging.
• Production of fusion genes for protein visualization in living cells or determination of protein-protein-interactions.
• Expression of a fusion protein mirroring physiological expression in its natural genetic context.

## Graphical abstract

**Figure:**
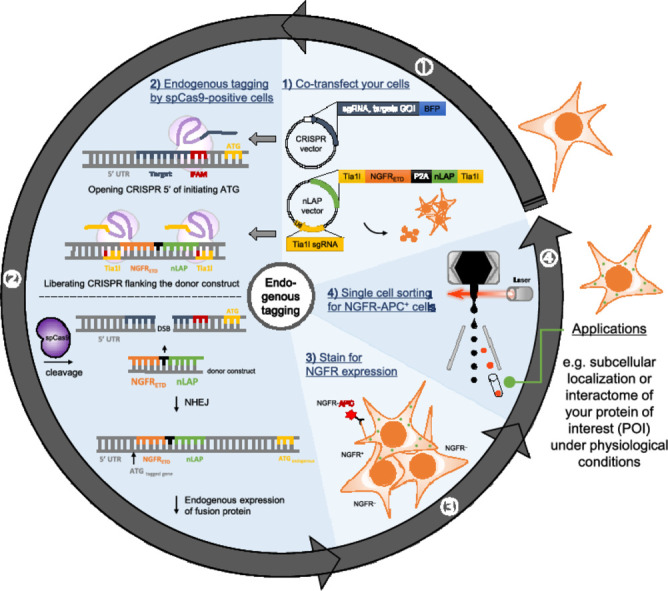

Specifications table

**Table:** 

Subject area	Biochemistry, Genetics and Molecular Biology
More specific subject area	Gene Editing
Name of your method	Selection-free endogenous tagging of cell lines
Name and reference of original method	CRISPR/Cas9-mediated generic protein tagging in mammalian cells, Thöne, F.M.B.; Kurrle, N.S.; von Melchner, H.; Schnütgen, F. CRISPR/Cas9-mediated generic protein tagging in mammalian cells. *Methods* 2019, *164*–*165*, 59–66. doi:10.1016/J.YMETH.2019.02.018
Resource availability	Vectors are made available via Addgene.org

## Introduction

Despite the tremendous progress in research, there is still a huge demand to explore the molecular mechanisms that explain the cellular processes involved in disease development. In particular, the analysis of complex signal transduction pathways in a native context is required for the understanding of cell physiology and pathophysiology. To achieve this, labeling of one single protein within this pathway with a fluorescent tag is one of the most straightforward and most successful applications of tag-based proteomics.

Such fluorescent fusion proteins not only allow, depending on the expression level, the tracing of proteins within living cells or organisms, but also the dissection of its protein-protein interactions that play a key role in virtually all biological processes involving proteins. However, it can be challenging to create fluorescent fusion proteins that retain all of the native properties of the protein-of-interest (POI). Potential overexpression artifacts and misregulated transcripts that confound subsequent results can be avoided by integrating the fluorescent tags directly into the respective endogenous genomic loci. Classically this is achieved via homologous recombination, but its inefficiency and high labor demand, particularly in somatic or non-dividing mammalian cells, impedes this approach. With the emergence of CRISPR/spCas9 genome editing tools, tagging strategies based on the non-homologous end joining (NHEJ) gene repair system have been developed, bypassing the time-consuming and labor-intensive generation of targeting constructs for homologous recombination, and enabling the production of endogenously tagged proteins much more easily and rapidly. As previously described in Thöne *et al.* (2019) [1], the CRISPR/spCas9 gene editing tool in combination with a generic tag-donor plasmid has been used very successfully to achieve endogenously targeted protein modifications coupled to antibiotic resistance markers for mammalian reporter cell selection.

Here, we provide an extended version of this strategy in which tagging of the POI is coupled to the expression of the surface marker Nerve Growth Factor Receptor (NGFR, LNGFR, CD271, p75NTR), which can be used to purify modified cells normally lacking NGFR expression with high purity and accuracy. To avoid NGFR-mediated signal transduction, a cytoplasmically truncated version preserving only the extracellular and the transmembrane domains (ETD) of the receptor is used [2]. Following this devised strategy, we employed a CRISPR/spCas9 expression vector and our previously published set of donor plasmids in which we have replaced the hygromycin-resistance cassette with the ETD of NGFR. These novel donor plasmids (pGTag NGFR EGFP nLAP) now encode the NGFR_ETD_-P2A-EGFP-encoding LAP tag [3], harboring the Enhanced Green Fluorescent Protein (EGFP)-containing localization and affinity purification (LAP) tag for N-terminal (nLAP) tagging. An equivalent version of a set of generic C-terminal tagging vectors was also constructed, here consequently in the reverse order of the respective cassettes (pGTag NGFR EGFP cLAP). The nLAP tag reported here consists of the EGFP for localization and immunopurification, the PreScission protease cleavage site for potential removal of the sterically challenging EGFP, the S-Peptide for the ability to perform a second affinity purification step, a TEV (tobacco etch virus) protease cleavage site for a second native elution step, and a FLAG tag for generic detection of the respective products [3]. The bicistronic co-expression of NGFR allows direct high purity FACS/MACS-based isolation of successfully targeted cells expressing endogenously EGFP-tagged proteins. Marking of cells using suitable fluorophore-coupled antibodies directed against NGFR, which is independently present as a surface marker on the cell due to the P2A site, circumvents thereby the tedious selection via an antibiotic resistance, which typically generates, due to the low endogenous tagging efficiency, an enormous number of dead cells often dragging the small number of successfully modified reporter cell clones with them to death. Moreover, fluorophore-coupled and high affinity anti-NGFR antibodies can subsequently be used for staining of successfully tagged cells, emitting unique signals that are distinctly different from the background and potentially appearing autofluorescence. By combining this downstream staining strategy with the surface marker-mediated FACS/MACS isolation, we can use this method in future to modify almost any cell line, especially in regard to suspension cells where the above-mentioned effort in antibiotic selection and subsequent single clone cultivation without the described adverse effect is particularly difficult to perform.

**Fig. 1:**
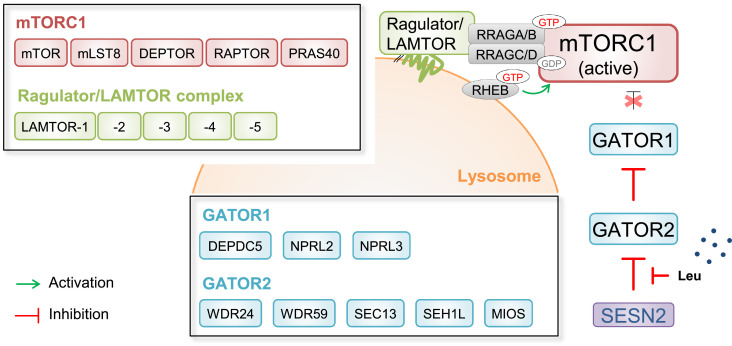
Leucine-dependent activation of mTORC1 at the lysosomal membrane. Leucine (Leu) is bound by sensing protein SESN2 and impedes its inhibition of GATOR2. The resulting in turn inhibitory function of GATOR2 disables the GTPase-activating protein (GAP) activity of GATOR1 enabling mTORC1 to be recruited to the lysosome by RRAG GTPases within the Ragulator/LAMTOR complex. At the lysosome, mTORC1 is activated by RHEB GTPase. Abbreviations: DEPDC5, DEP Domain Containing 5; DEPTOR, DEP Domain-Containing mTOR-Interacting Protein; GATOR, GAP activity towards RAG; GDP, Guanosine diphosphate; GTP, Guanosine triphosphate; LAMTOR, Late Endosomal/Lysosomal Adaptor And MAPK And MTOR Activator; Leu, leucine; MIOS, Meiosis Regulator For Oocyte Development (Homolog (Drosophila)); mLST8, Mammalian Lethal with SEC13 Protein 8; mTOR, mechanistic Target Of Rapamycin; NPRL2/3, Nitrogen Permease Regulator-Like Protein 2/3; PRAS40, Proline-Rich AKT Substrate Of 40 kDa; RAPTOR, Regulatory-Associated Protein Of mTOR; RHEB, RAS Homolog Enriched In Brain; RRAGA/B/C/D, RAS Related GTP Binding A/B/C/D; SEC13, SEC13 Homolog, Nuclear Pore And COPII Coat Complex Component; SEH1L, SEH1 Like Nucleoporin; SESN2, Sestrin-2; WDR24/59, WD Repeat Domain 24/59.

To assess the functionality of this promising strategy, we first used this toolkit to tag WD Repeat Ddomain 59 (WDR59), a member of the mammalian Target of Rapamycin Complex 1 (mTORC1)-regulatory GATOR2 complex in spCas9-expressing HEK293T cells (Lenti-X^TM^ 293T spCas9^+^). Proteomic validation of the NGFR_ETD_-nLAP-tagged protein showed that the WDR59 fusion protein replicated the protein-protein interactions of its native counterpart. In detail, the central metabolic hub of mammalian cells, mTORC1, is regulated by amino acids through the GATOR-RRAG GTPase axis. According to current knowledge, in the absence of amino acids, amino acid-sensing proteins (e.g. Sestrin-2 (SESN2) for leucine-sensing [4]) bind in an inhibitory manner to the pentameric GATOR2 complex, consisting of WDR59, WDR24, Meiosis Regulator For Oocyte Development (MIOS), SEC13 Homolog, Nuclear Pore And COPII Coat Complex Component (SEC13), and SEH1 Like Nucleoporin (SEH1L). The binding of these amino acid-sensing proteins to GATOR2 prevents GATOR2-mediated inhibition of GTPase activating protein (GAP) activity toward Rags 1 (GATOR1). GATOR1 activity in turn, impedes mTORC1 recruitment by lysosomal, then GDP-bound, RRAGA/B GTPases during amino acid scarcity. In the presence of amino acids, e.g. leucine, the respective cytosolic leucine sensing protein SESN2 is bound by leucine, detaches from GATOR2, which in turn impedes GATOR1 activity. Inactivation of GATOR1 instantaneously promotes mTORC1 recruitment by lysosomal GTP-bound RRAGA/B GTPases and its activation (Fig. 1) (described in more detail in Seibert *et al*. (2021) [5]).

The amino acid-/ leucine-dependent interaction between SESN2 and GATOR2 was used to validate the endogenous metabolic function of nLAP-tagged GATOR2 member WDR59. Because the functionality of this signaling hub requires a variety of protein-protein interactions and thus these different complexes must possess a variety of interaction domains, we considered a protein of the GATOR2 complex to be the ideal target protein to test the functionality of this endogenous tagging strategy. Here we describe the individual components of the endogenous tagging toolkit using WDR59 as an example and give guidance for its use regarding the cell selection using the NGFR_ETD_ surface marker.

## Materials

### Cell culture and growth/starvation medium

Lenti-X^TM^ 293T cells (TaKaRa #632180) pretransduced with pLentiCas9-Blast (Addgene #52962) to stably express spCas9, see Fig. 3B (in following referred to as HEK spCas9^+^ cells) were cultivated at 37°C with 5% CO_2_ in 10% FCS high glucose DMEM medium: 450 ml DMEM (1x) high glucose (Gibco™, Thermo Fisher, #41965-039), supplemented with 50 ml FCS (Sigma-Aldrich, #F7524) and 100 U/mL penicillin/ 100 µg/mL streptomycin (1x P/S) (stock 100x: 10,000 U/mL penicillin, 10 mg/mL streptomycin, Gibco™ Penicillin-Streptomycin #15140122).

For general cell passage, cells reaching 80-90% confluency were washed with 1x PBS (Gibco™ DPBS, without Ca^2+^, Mg^2+^, #14190-094) and detached from culture plates with 1x Trypsin-EDTA solution (Sigma-Aldrich, #59418C, diluted with 1x PBS). Every third day, cells were resuspended in fresh supplemented DMEM and reseeded into fresh culture plates at a ratio of 1:10 and cultivated until 80-90% confluency was again achieved.

For starvation experiments, amino acid-free RPMI 1640 medium (powder, w/o amino acids, sodium phosphate, USBiological, #R8999-04A) and leucine-free RPMI 1640 medium (powder, w/o glutamine, leucine, USBiological, #R8999-03) was dissolved in DNase/RNase-free distilled water (ddH_2_O, Invitrogen, #10977-035) according to the manufacturer's instructions and supplemented with 2 g/L sodium bicarbonate (Sigma, #S5761) and 1x P/S. In amino acid-free medium, 800mg/L sodium phosphate (Sigma, #S5136) was added; in leucine-free medium 2 mM glutamine (Sigma, #G7513) was added. Cell culture under starved conditions was always performed in presence of 1% (v/v) dialyzed FCS (dFCS) (#26400 Gibco™, Thermo Fisher) with a cell confluency of 70-80%. Subsequent stimulations with all amino acids were performed by using RPMI 1640 medium (#21875034 Gibco™, Thermo Fisher) supplemented with 1% dFCS and 1x P/S. For leucine-stimulation, leucine (Sigma, #L8912) was dissolved in 1 M HCl to concentration of 380 mM (1000x final concentration) and added to leucine-free RPMI 1640 medium, supplemented with 1% dFCS and 1x P/S. In all media used, pH of 7.4 was adjusted using 1 M NaOH (AppliChem #1432). Starvation and stimulation times were as indicated in results.

### Molecular biology reagents

For cloning vectors:

• Restriction enzyme (BsmBI-v2) (New England Biolabs, #R0739L)
• Restriction enzyme (ClaI) (New England Biolabs, #R0197S)
• Restriction enzyme (XmaI) (New England Biolabs, #R0180S)
• Antarctic Phosphatase (New England Biolabs, #M0289L)
• T4 DNA ligase (New England Biolabs, #M0202L)
• Q5® High-Fidelity DNA Polymerase (New England Biolabs, #M0491L)

For transfecting HEK spCas9^±^ cells:

• Lipofectamine 3000 Reagent (Invitrogen™, #L3000015)

For PCR-based validations:

• DreamTaq™ Green DNA Polymerase (Thermo Scientific, #EP0711)
• DreamTaq™ Green buffer (Thermo Scientific, #K1081)
• For CoIP experiments and Western blot-based validations:
• ChromoTek GFP-Trap® Magnetic Agarose (#gtma-20)
• ChromoTek Binding control magnetic agarose (#bmab-20)

### Oligonucleotide sequences for WDR59-specific sgRNA

All oligonucleotides were obtained from Sigma-Aldrich. Uppercase letters indicate the gene-specific sequences; lowercase letters indicate the overhangs required for cloning:

Sense sgRNA targeting WDR59:

5’-caccgCGCCGTCCTGGGGCCGCGG-3’

Antisense sgRNA targeting WDR59:

5’-aaacCCGCGGCCCCAGGACGGCGc-3’

### PCR primer oligonucleotide sequences

All primers were obtained from Sigma-Aldrich.

**Table:** 

Generic tag forward primer (TF)	CA206: 5’-CAGCTGCTGCTAAATTCGAG-3’
Generic tag reverse primer (TR)	CA207: 5’-CACCGCTGTGTGTGTACAGG-3’
Endogenous forward primer WDR59 (EF)	CA212: 5’-GACTACAACTCCCGGCAGAG-3’
Endogenous reverse primer WDR59 (ER)	CA213: 5’-GTAGGGAGCCCCGAAACTC-3’

### Buffers for protein biochemistry

Coimmunoprecipitation buffer (CoIP) buffer:

• 10 mM Tris-HCl pH8 (Carl Roth GmbH, #9090.3)
• 150 mM NaCl (Riedel-de-Haёn, #31434)
• 5 mM EDTA pH8 (Solution, PanReac AppliChem, #A4892,0500)
• 0.5% Triton X-100 (Fluka, #93420)
• 60 mM Octyl-β-D-glucopyranoside (Sigma-Aldrich, #O8001)

Buffer is supplemented with Protease Inhibitor Cocktail (cOmplete^TM^, Roche Diagnostics GmbH, #05056489001), 1 mM NaF (Sigma-Aldrich, #S7920), 1 mM Na_3_VO_4_ (Sigma, #S6508)

SDS-Lysis buffer:

• 100 mM Tris HCl pH 8.0 (Carl Roth GmbH, #9090.3)
• 150 mM NaCl (Riedel-de-Haёn, #31434)
• 10 mM EDTA pH 8.0 (Solution, PanReac AppliChem, #A4892,0500)
• 10% SDS (MP Biomedicals #04811033-CF)

Lowry Assay (Protein concentration measurement at 650–750 nm):

• DC^TM^ Protein Assay Reagent B (#500-0114), - Reagent A (#5000113), Bio-Rad Laboratories), used according to the manufacturer's instructions

SDS Loading Dye (4x final concentration) (for Western blot sample preparation):

• 250 mM Tris HCl pH 6.8 (Carl Roth GmbH, #9090.3)
• 8% SDS (MP Biomedicals #04811033-CF)
• 40% Glycerol (Carl Roth GmbH, #3783.1)
• 0.2% Bromphenol blue (Sigma-Aldrich, #B0126)

### Antibodies (Western Blot/ Flow cytometry/ FACS)

For Western blot detection all antibodies were diluted 1:1,000 in TBS buffer B (Zytomed Systems, #ZUC066), containing 0.05% (v/v) Tween-20 (Carl Roth GmbH, #9127.1) and 0.5% (v/v) NaN_3_ (Sigma, #S2002), except for the GAPDH antibody which was applied in a 1:20,000 dilution. The following primary antibodies were used:

• β-Actin (Cell Signaling (13E5), #4970)
• GFP (Roche Diagnostics GmbH, #11814460001)
• GAPDH (Abcam (6C5), #ab8245)
• NPRL3 (Invitrogen, #PA5-78245)
• SESN2 (ProteinTech, #10795-1)
• MIOS (Cell Signaling (D12C6), #13557)
• WDR59 (Cell Signaling (D4Z7A), #53385)
• WDR24 (ProteinTech, #20778-1-AP)
• 4EBP1 (Cell signaling, #9452)
• Phospho-4EBP1 (T37/T46) (Cell signaling (236B4), #2855)
• FLAG® M2 (Sigma-Aldrich, #F1804)

The following secondary antibodies (diluted 1:10,000) were used:

• HRP-AffiniPure Goat Anti-Mouse IgG (H+L) (JacksonImmunoResearch, #115-035-146)
• HRP-AffiniPure Goat Anti-Rabbit IgG (H+L) (JacksonImmunoResearch, #111-035-003)

For FACS-staining

• Anti-CD271 (LNGFR) Antibody, anti-human/mouse, APC REAfinity™ (Miltenyi Biotec, #130-116-497)

### Flow cytometry/ FACS instruments

BD FACS Aria^TM^ II | High Sensitivity Flow Cytometry Cell Sorter

BD LSRFortessa^TM^ Cell Analyzer – Flow Cytometer

### Software

• BD FACSDiva™ Software, FlowJo™ v7.6.5 Software (BD Life Sciences)
• ImageJ Software [6]
• Benchling [Biology Software] (2022) retrieved from https://benchling.com

## Methods Details

### The generic protein tagging method

**Fig. 2:**
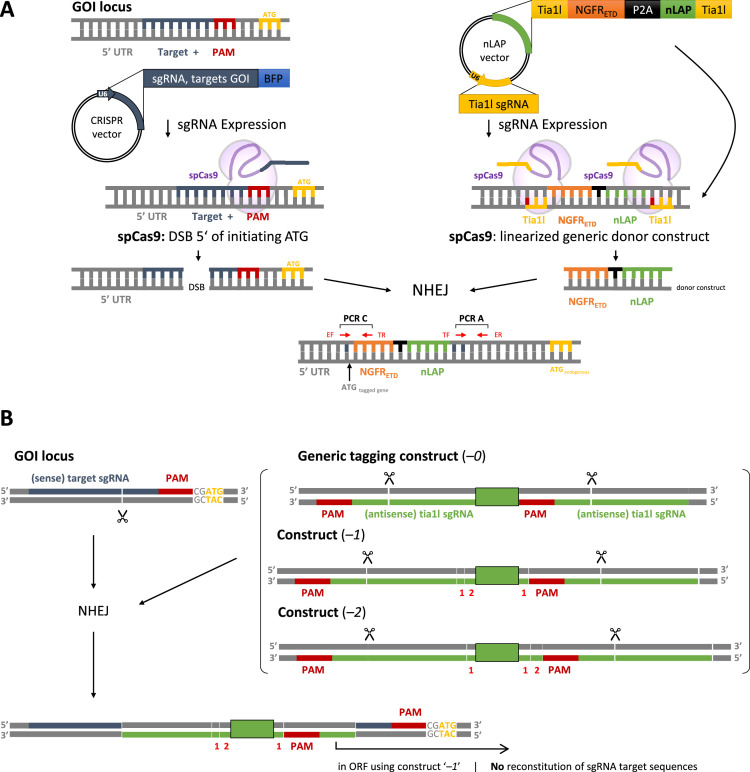
Homology-independent CRISPR/spCas9-mediated endogenous tagging strategy. **A** Schematic representation of the tagging strategy. spCas9^+^ cells are co-transfected with a vector expressing a target locus-specific sgRNA (here: pLentiCRISPRv2ΔspCas9_BFP) and an nLAP vector expressing the generic donor construct consisting of the NGFR_ETD_-P2A-nLAP cassette flanked by *tia1l* sites and a U6 promoter-driven *tia1l* sgRNA sequence. Expression of spCas9 results in release of the NGFR_ETD_-P2A-nLAP tag from the donor plasmid and insertion into the target site, here within the 5’UTR of a GOI. The red arrows indicate the position of the primers used for PCR verification of the tag-endogenous gene junctions: EF/ER = endogenous forward/reverse, TF/TR = tag forward/reverse. **B** Locating the *tia1l* sites and endogenous target site on opposite strands prevents new formation of sgRNA target sequences. To retain the ORF, the target gene-determined correct donor construct must be used (here as an example: with one (1) additional base pair downstream of the nLAP tag). Numbers on the schematic strands indicate individual base pairs. The lengths of the DNA strands represent not the number of base pairs. The integrated NGFR-P2A EGFP donor cassette is indicated with a green box. **Abbreviations**: sgRNA, single guide RNA; NGFR, Nerve Growth Factor Receptor; P2A, 2A virus polyprotein cleavage sequence; nLAP, N-terminal protein localization and purification tag; GOI, gene-of-interest; NHEJ, non-homologous end joining; ORF, open reading frame; PAM, protospacer adjacent motif

As described by Thöne *et al*. (2019) [1] and Lackner *et al*. (2015) [7], the generic tagging method includes co-transfection of a donor plasmid and a target locus-specific sgRNA-expressing plasmid (Fig. 2A). To avoid transfection with the very large spCas9 expression vector, spCas9 pre-transduced Lenti-X 293T cells were used here (referred to as HEK spCas9^+^ cells).

The choice of the site for the target-specific sgRNA for N-terminal labeling of a protein is relatively arbitrary, but several points must be considered in the selection. First, it should be checked whether an in-frame stop codon exists in the 5′ UTR upstream of the initiation ATG. If this is the case, care must be taken to ensure that the gene-specific sgRNA is located between this in-frame stop codon and the initiation ATG codon, otherwise translation will be terminated after successful labeling at this stop codon. Other selection criteria are based on the on-target and off-target score of the potential sgRNA, which we determine using the benchling software package (Benchling [Biology Software] (2022) retrieved from https://benchling.com). After selecting the appropriate sgRNA sequence, the donor plasmid matching this sgRNA is chosen so that no stop codon is generated upon integration of the nLAP tag, the reading frame is maintained across the entire fusion gene and the sgRNA sequences are not reconstituted. Notice that with all the donor-vector sets we have constructed so far, it is also possible to tag downstream of the initiation ATG. The donor plasmid contains the nLAP tag corresponding to the target's reading frame flanked by *tia1l* sgRNA binding sites from zebrafish not present in mammalian cells. The liberation of the tag, via spCas9-mediated cleavage at the two *tia1l* sequences, is initiated by the expression of the *tia1l* sgRNA, which is encoded on the donor plasmid itself and driven by an U6 promoter. When the donor plasmid is co-transfected into spCas9-expressing mammalian cells together with a plasmid expressing an sgRNA specific for the endogenous target locus of the gene-of-interest (GOI), the target locus is sequence-specifically opened at the locus of choice and simultaneously the linear nLAP tag is released from the donor vector. Repair of the endogenous target locus is mediated through the NHEJ pathway but the presence of the linear nLAP tag results coincidentally in the incorporation of the tag in some cases, which subsequently become selectable. To prevent reconstitution of one of the sgRNA target sequences after tag insertion, which would lead to a new CRISPR/spCas9-mediated DNA double-strand break (DSB), we generated two donor plasmids for each reading frame, with either flanking *tia1l* sgRNA targets in sense (+) or antisense (-) orientation (Fig. 2B and C) and typically use the antithetical *tia1l* sgRNA binding sites relative to the target-specific sgRNA sequence.

Here, we describe a set of vectors that not only encode the nLAP tag itself, but additionally bicistronically express the truncated surface marker NGFR composed of the extracellular and the transmembrane domains (NGFR_ETD_) linked to the EGFP nLAP tag via a P2A polyprotein cleavage sequence. The co-expressed NGFR_ETD_, after successful tagging of the target protein, allows fluorescence activated cell sorting (FACS) with any commercially available fluorophore coupled to anti-NGFR antibodies. It should be mentioned here that antibody-mediated fluorescence via a surface marker typically generates significantly stronger signals than an EGFP that relies on the typically weak expression of the endogenous fusion protein. This advantage is used here to isolate correctly modified cells with high accuracy. As an alternative, the use of magnetic-labeled anti-NGFR antibodies allows also magnetic-activated cell sorting (MACS). Hence, with this strategy, the use and variable sorting of cells expressing endogenous protein tags, also other than EGFP, is possible.

### NGFR_ETD_-nLAP donor plasmids for protein tagging

To exchange the hygromycin resistance cassette for the cytoplasmically truncated NGFR from the existing set of pGTag Hygro EGFP nLAP donor vectors (Addgene #194305, #194306, #194307, #194320, #194321 and #194322) [1], the NGFR_ETD_ cassette from the vector PAULO [8] was amplified with reading frame-specific primers and inserted by ClaI/XmaI digestion into the existing vectors. The resulting vector was named pGTag NGFR EGFP nLAP, and all six different variants of the original vector (+0, +1, +2, -0, -1, and -2) were retained, comprising the respective orientations of the *tia1l* sgRNA binding sites and three different reading frames for each orientation (Addgene #194314, #194315, #194316, #194505, #194506 and #194507). Corresponding C-terminal tagging vectors were also constructed (Addgene #194308, #194309, #194310, #194311, #194312, #194313, #194508, #194509, #194510, #194511, #194512, #194513).

### Cloning of locus specific targeting vectors

We designed all sgRNAs using the Benchling software package (Benchling [Biology Software] (2022) retrieved from https://benchling.com). For expression of the target protein-specific sgRNA, we used the commercially available pLentiCRISPRv2 plasmid (Addgene #52961), in which spCas9 and the cDNA encoding the puromycin resistance cassette was replaced by a TagBFP-encoding cDNA. Successful transfection with this target plasmid can thus be verified by BFP expression, both under the microscope or alternatively via flow cytometry. As transfection efficiency is strictly dependent on plasmid sizes, we herein applied HEK spCas9^+^ cells and used therefore the pLentiCRISPRv2ΔspCas9_BFP plasmid.

The cloning of target locus-specific sgRNAs in pLentiCRISPRv2ΔspCas9_BFP was performed according to the Golden Gate protocol [9]:

(a) Anneal oligonucleotides complementary to preselected targets that are upstream of the initiating ATG by adding 5 µl of each oligonucleotide (100 µM) to 40 µl ddH_2_O. Incubate at 98°C for 5 min and cool down for 10 min at room temperature to achieve annealing.
(b) Prepare a Golden Gate reaction mix with a cumulative volume of 20 µL (ddH_2_O):

• 150 ng pLentiCRISPRv2ΔspCas9_BFP
• 5 µL annealed oligonucleotide mix
• 2 µl 10X ligase buffer,
• 1 µl T4 DNA ligase
• 1 µl of BstBI restriction enzyme

(c) Perform Golden Gate reaction in a thermocycler programmed as follows:

• Initial digestion at 37°C for 5 min followed by
• 10 cycles: 16°C for 10 min (ligation)

37°C for 15 min (digestion) followed by

• Denaturation at 80°C for 5 min.

(d) Use the Golden Gate reaction product directly for transfection into competent E. coli (DH5α, Invitrogen^TM^, #18265017).

### Co-transfection of HEK spCas9^+^ cells with targeting and donor-constructs

Select the donor plasmid to preserve the reading frame of the target gene. This is mandatory because after correct tagging, the ATG of the NGFR_ETD_ cassette determines the reading frame of the tagged allele and depending on the position where the endogenous CRISPR generates the DNA double strand break, the reading frame to the original initiating ATG of the endogenous gene must be preserved. This was achieved by constructing three different reading frames of the donor vectors (NGFR “0,” “1” or “2”). For the design of the endogenous tagging strategy, assume a seamless, indel-less integration. In addition, the donor plasmid should be chosen so that the CRISPR *tia1l* sites are on the opposite strand of the respective CRISPR target site (construct “+” (sense) in case the endogenous CRISPR target site is on the antisense strand, or “–” (antisense) in case the endogenous CRISPR target site is on the sense strand) (see Fig. 2B and C). Using equimolar amounts of donor- and targeting vectors gave the best results. In the case of endogenous WDR59-NGFR_ETD_-nLAP tagging, NGFR “-2” was used.

Co-transfection protocol for adherent HEK spCas9^±^ cells:

(a) Seed 1.5×10^6^ HEK293T spCas9^+^ cells into a 10 cm cell culture dish. Make sure that the cells are in exponential growth state and evenly distributed over the entire surface of the dish at a density where they can remain for 4-5 days to ensure endogenous tagging. Avoid passaging cells after transfection, especially by using trypsin, as this would also digest the surface exposed NGFR_ETD_. Incubate at 37°C overnight.
(b) On the next day, prepare the transfection mix (here, Lipofectamine™ 3000, Invitrogen, #L3000001), according to the manufacturer's instructions:

**Table:** 

**Solution A:**	**Solution B:**
+ 20 µL P3000	+ 22,5 µL Lipofectamine3000
(2 µL per µg DNA)	
+ DNA (10 µg; 5 µg each)	+ 500 µL DMEM medium
+ 500 µL DMEM medium	(without FCS and antibiotics)
(without FCS and antibiotics)

• Vortex both solutions briefly, incubate 5 min at room temperature
• Combine solution A and B and incubate transfection mix for 20 min at room temperature

(c) Change medium of cells carefully to fresh 7 mL growth medium (DMEM, supplemented with 10% FCS and 1 x Penicillin/Streptomycin)
(d) Add the transfection mix dropwise to the cells and spread it evenly across the dish.
(e) Incubate for 1 h at 37°C. Shake plates gently before re-incubating overnight
(f) Change medium to fresh 10 mL growth medium and expand cells to 95-100% confluence.
(g) On day 3 after transfection, it is possible to check for BFP-expression indicating uptake of targeting plasmids

### NGFR_ETD_-APC-staining and subsequent fluorescence-activated cell sorting (FACS)

Prepare a sterile solution containing 1x phosphate-buffered saline (1x PBS), pH 7.2, 0.5% bovine serum albumin (BSA), and 2 mM EDTA pH 8.0

(a) After approximately 4-5 days, remove medium of ∼100% confluent cells and wash carefully with 1x PBS
(b) Remove all 1x PBS and add 3 mL 1x PBS/BSA/EDTA for the now typically available 15×10^6^ cells. Do not use trypsin because it degrades the surface exposed NGFR_ETD_ marker on the cell's plasma membrane.
(c) Rock gently until cells are in solution, transfer all cells into a sterile tube
(d) Rinse plate with 1x PBS/BSA/EDTA if necessary.
(e) Resuspend cells thoroughly and stain by adding 10µL (NGFR) Antibody, anti-human, (APC, or any other coupled fluorophore, e.g. Miltenyi Biotec, #130-116-497). The amount of antibody is highly dependent on the amount of positive cells and may need to be adjusted.
(f) Mix well and incubate for 30 minutes in the dark at 4°C.
(g) Wash cells by adding 3 mL of buffer and centrifuge at 300×g for 5 minutes. Aspirate supernatant completely.
(h) Resuspend cell pellet in appropriate volume of 1x PBS/BSA/EDTA for sorting.
(i) Sort for the highest 5% of NGFR-APC positive cells using stained wild type (WT) cells as a control. Stain WT cells equally (use 500,000 cells in 100 µL 1x PBS/BSA/EDTA with 1 µL (NGFR) Antibody, anti-human, (APC))
(j) Sub-cultivate sorted cells at appropriate density in growth medium (DMEM, supplemented with 10% FCS and 1 x Penicillin/Streptomycin
(k) Examine plates daily until single colonies become visible by eye.

### Single clone cultivation

Carefully separate individual colonies by gently scraping them off the plate using a 200 µL pipette and add them individually into the wells of a 48-well dish containing 500 µL growth medium. To obtain single cells of each colony, after one day of incubation at 37°C, wash with 1 mL 1x PBS and add 50 µL 1x trypsin per well. After 3 min of incubation at room temperature, the cells of each colony can be individualized with 500 µL of growth medium by carefully pipetting up and down with a 200-µL pipette. The cell suspension is further cultivated until reaching 70% confluence. Note that incubation times may vary from clone to clone.

Distribute the cells of each clonal cell line into three wells of a 24-well plate using the same procedure and further expand these cells until an appropriate cell number of each clonal cell line is obtained. Use one well of a 6 well plate for DNA analysis (PCR of integration sites), another well for Western blot analysis and the final well for further cultivation and freezing.

### Molecular analysis of reporter cell clones

The correct integration of the NGFR_ETD_-nLAP tag in individual clones is identified by PCR amplification of the junction between tag and target gene and subsequent sequencing. Primers are designed (see 2.4) to amplify the 3’ junction of the tag (nLAP / target gene (PCR A)), as well as the 5’ junction of the tag (between 5’-UTR of the target gene and the 5′-end of the tag (PCR C)). For a schematic representation of the primer binding sites, see Fig. 2A, red arrows. Tag integrations generate PCR products at the 5’-junction, as well as at the 3’-junction of the tag. Notice that a correct tag insertion is only given, when the tag is in frame with the open reading frame (ORF) of the endogenous target gene to allow correct transcription of the tagged allele. This is normally obtained when a seamless, indel-less integration of the tag has been achieved. An insertion or deletion of whole triplets is permitted, but here care must be taken that no in frame stop codon is generated. Additionally, the amplification of the WT target allele is performed to check whether the WT allele (in case of polyploid cells WT alleles) is still intact, or if expression-disturbing indels were created here (PCR B - control PCR). Note that unlike the gene specific primers, the primers binding to the tag are generic and can be used for any target site.

Subsequently, confirm nLAP-tagged protein expression in selected and PCR-confirmed cell clones by Western blot analysis as well as by flow cytometry.

#### PCR of integration sites

(a) Extract and purify genomic DNA (gDNA) of different clones by Phenol/Chloroform/Isoamylalkohol method or any other standard protocol
(b) Use 100 ng gDNA per PCR

**Table:** 

DreamTaq^TM^ Green Buffer:	5 µL
NTPs [10 mM]:	2 µL
Primer forward [10 mM]:	2.5 µL
Primer reverse [10 mM]:	2.5 µL
DreamTaq^TM^ Green:	0.3 µL
DNA:	100 ng
ddH_2_O:	ad 50 µL

(a) Combine forward and reverse primers according to PCR A, B or C
(b) PCR program: Run initial denaturation at 95°C for 1 min, followed by 32 cycles at 95°C for 30 s (denaturation), 57°C for 30 s (annealing), 72°C for 30 s (elongation) followed by a final elongation at 72°C for 10 min. Annealing temperatures are primer dependent. The melting temperatures of all primers used here are adjusted to 60°C.

#### Western blot of tagged protein expression/ flow cytometry

(a) Lyse cell clones for whole cell lysates by adding SDS lysis buffer directly to adherent cells. Scrape off cells and transfer them into 1.5 mL tubes. Measure protein concentration (Lowry Assay) and run standard Western blotting using anti-GFP antibodies to visualize nLAP-tagged protein. Re-probing blots with high affinity antibodies for native proteins allows identification of both, tagged and native proteins.
(b) For flow cytometry, collect cells by trypsinization, wash cells with 4 mL 1x PBS and transfer a small aliquot to a flow cytometry tube. GFP signals can be obtained using LSRFortessa FACS machine (or equivalent) exciting GFP at 488 nm and GFP-detection through 530 nm bandpass filter. Note that NGFR-APC staining (described above) is inapplicable after trypsinization.

### Analysis of protein-protein interactions using generated reporter cell lines

Endogenously tagged proteins vastly simplify interaction studies. Endogenous fusion with GFP allows the use of high affinity anti-GFP antibodies instead of often less well-suited antibodies directly targeting the endogenous protein. The use of GFP specific nanobodies (ChromoTek GFP-Trap®) coupled to magnetic agarose beads allows easy co-immunoprecipitation of GFP fusion proteins and interaction partners. To check for interaction partners of nLAP-tagged WDR59:

(a) Seed 5×10^6^ HEK WDR59-NGFR_ETD_-nLAP cells onto a 10 cm dish containing 10 mL growth medium and grow cells until ∼80% confluent
(b) Wash cells carefully with cold 1x PBS and remove PBS completely before adding 400µL CoIP buffer (supplemented) per plate
(c) Scrape off cells, transfer them in centrifugation tubes, incubate 30 min on ice, centrifuge cell lysate at 15,000 rpm (2,767x g) and measure protein concentrations of the supernatant (Bradford assay).
(d) Incubate 1,000-3,000 µg of protein in presence of 25 µL GFP-Trap® magnetic agarose beads overnight at 4°C on a rotating wheel. Use the same volume of binding control magnetic agarose beads (ChromoTek®) as control. Note that the appropriate protein concentration and the corresponding amount of beads depend on the expression of the tagged protein and needs to be adjusted to improve the CoIP result.
(e) Wash beads 5 times with CoIP buffer using a magnet. Number of washing steps may increase with higher protein use.
(f) Boil beads in presence of 10-15 µL 4x SDS loading dye at 95°C for 5 min, run SDS-PAGE and transfer to blotting membrane.

## Representative Results

### Tagging of mTORC1-regulating GATOR2 complex subunit WDR59 in HEK spCas9^+^ cells and subsequent sorting of correct cells using anti-NGFR-APC antibody staining

As previously demonstrated in Thöne *et al.* (2019)[1], tagging efficiencies vary, because they are dependent on the particular target locus. To demonstrate that allele-specific tagging and subsequent selection of successfully protein-tagged cells using NGFR_ETD_ surface expression is suitable for protein-protein interaction studies, we selected a protein whose native counterpart is known to be incorporated into large protein complexes. Here, we chose a subunit from the mTORC1-regulatory GATOR2 complex: WDR59. GATOR2 indirectly activates mTORC1 in an amino acid- and leucine-dependent manner and serves as a metabolic modulator upstream of mTORC1 depicting an excellent example to validate the selection-free endogenous tagging strategy.

**Fig. 3:**
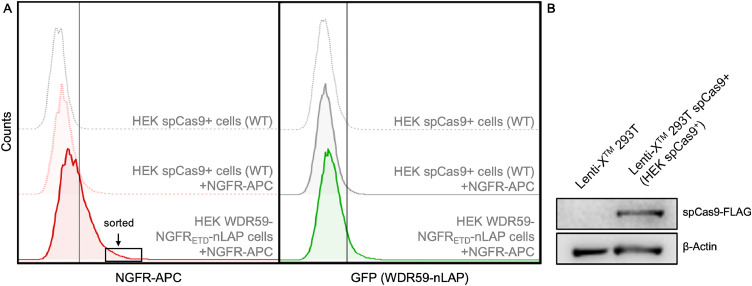
Comparison of anti-NGFR APC flow cytometry versus GFP autofluorescence. **A** Sorting of HEK WDR59-NGFR_ETD_-nLAP cells by anti-NGFR-APC staining. Comparison between NGFR-APC signal and endogenous GFP expression of co-transfected cells before NGFR-APC sorting. Left panel: HEK (Lenti-X 293T) spCas9^+^ cells (wild type (WT)) with and without NGFR-APC staining. After successful tagging, HEK WDR59-NGFR_ETD_-nLAP cells, stained with APC-conjugated anti-NGFR antibodies, showed high expression of NGFR, of which the highest 5% NGFR-APC positive cells were sorted and sub-cultivated in single clones. In contrast, endogenous GFP expression (right panel) is weaker and rather unsuitable to guarantee sorting with appropriate purity and accuracy. **B** Western Blot for pre-expressed spCas9-FLAG in the Lenti-X^TM^ 293T cells used for endogenous tagging of WDR59. β-Actin serves as loading control. The flow cytometry results were analyzed using FlowJo™ v7.6.5 Software (BD Life Sciences).

The first step in obtaining cells with correct nLAP-tagged WDR59, was to stain co-transfected cells with APC conjugated anti-NGFR antibodies and subsequently sort for highest APC abundance (see Fig. 3A, left panel). The necessary expression of spCas9 in the Lenti-X^TM^ 293T cells used was previously verified by Western blot Fig. 3B). NGFR-APC-sorted cells were then used to generate single cell clones, to guarantee a pure, genetically identical, clonal population of WDR59-NGFR_ETD_-nLAP expressing HEK spCas9^+^ cells.

As previously mentioned, endogenous GFP expression could also be used for sorting correct cell clones (Fig. 3, right panel), but by endogenously tagging of weakly expressed proteins, the coupled GFP expression may also be too weak to guarantee sorting with appropriate purity and accuracy. Sorted cells were cultured in small colonies which were separated from each other to generate clonal populations for subsequent single-clone analysis for correct tagging.

### Analysis of NGFR_ETD_-nLAP-tagged WDR59 in HEK WDR59-NGFR_ETD_-nLAP single cell clones

In total, twenty HEK WDR59-NGFR_ETD_-nLAP clones were isolated and analyzed by PCRs. Of these twenty WDR59 clones, a correct 3′-junction of the nLAP tag could be shown in three clones without frameshift mutations of the ORF (PCR A, see Table 1). One clone (B1) had a deletion of 6 base pairs (bp) (loss of two amino acids) upstream of the junction. However, due to the divisible number of bp by three, a correct ORF can also be assumed, so that all four clones promise a correct expression of the WDR59-GFP fusion protein. The remaining sixteen clones failed to generate a PCR product of the 3’-juncton of the nLAP tag.

**Table 1:** Successful sequencing of nLAP-tagged *WDR59* – PCR A refers to NGFR_ETD_-WDR59 junction; PCR B refers to WT allele amplification; PCR C refers to 5’UTR-NGFR_ETD_ junction. Bold ATGs are endogenous in-frame start codons of *WDR59* (PCR A, B) or *NGFR* (PCR C), underlined bases mark insertions, dash lines mark deletions. Indels are indicated in clone names.

WDR59 clone	PCR	Sequence *5’ (NGFR (nLAP))*	← junction *(WDR59) ’3*
(reference)	A	CCGACGACCTGCCCAGGGCCTCGTTCCTGGA	CGG**CGG**CCGGGCGGGGGAG**ATG**GCGGCGCGATG
B1 *(-6)*	A	CCGACGACCTGCCCAGGGCCTCGTTC—–	-GG**CGG**CCGGGCGGGGGAG**ATG**GCGGCGCGATG
B3 *(0)*	A	CCGACGACCTGCCCAGGGCCTCGTTCCTGGA	CGG**CGG**CCGGGCGGGGGAG**ATG**GCGGCGCGATG
D4 *(0)*	A	CCGACGACCTGCCCAGGGCCTCGTTCCTGGA	CGG**CGG**CCGGGCGGGGGAG**ATG**GCGGCGCGATG
D5 *(0)*	A	CCGACGACCTGCCCAGGGCCTCGTTCCTGGA	CGG**CGG**CCGGGCGGGGGAG**ATG**GCGGCGCGATG
		*5’ (WDR59)*	*(WDR59) ’3*
WT	B	GGCGGGGTGGGAGGGCGCCGTCCTGGGGCCG	CGGCGGCCGGGCGGGGGAG**ATG**GCGGCGCGATG
B1 *(-9)*	B	GGCGGGGTGGGAGGGCGCCGTCCTGGGGCC-	——–GGGCGGGGGAG**ATG**GCGGCGCGATG
B3 *(+1)*	B	GGCGGGGTGGGAGGGCGCCGTCCTGGGGCCGC	CGGCGGCCGGGCGGGGGAG**ATG**GCGGCGCGATG
D4 *(0)*	B	GGCGGGGTGGGAGGGCGCCGTCCTGGGGCCG	CGGCGGCCGGGCGGGGGAG**ATG**GCGGCGCGATG
D5 *(+1)*	B	GGCGGGGTGGGAGGGCGCCGTCCTGGGGCCGA	CGGCGGCCGGGCGGGGGAG**ATG**GCGGCGCGATG
		*5’ (WDR59)*	*(NGFR (nLAP)) ‘3*
(reference)	C	GGCGGGGTGGGAGGGCGCCGTCCTGGGGCCG	GAGGTTCCCGACATACCTAAGTACCACC**ATG**GG
B1 *(0)*	C	GGCGGGGTGGGAGGGCGCCGTCCTGGGGCCG	GAGGTTCCCGACATACCTAAGTACCACC**ATG**GG
B3 *(0)*	C	GGCGGGGTGGGAGGGCGCCGTCCTGGGGCCG	GAGGTTCCCGACATACCTAAGTACCACC**ATG**GG
D4 *(0)*	C	GGCGGGGTGGGAGGGCGCCGTCCTGGGGCCG	GAGGTTCCCGACATACCTAAGTACCACC**ATG**GG
D5 *(0)*	C	GGCGGGGTGGGAGGGCGCCGTCCTGGGGCCG	GAGGTTCCCGACATACCTAAGTACCACC**ATG**GG

**Fig. 4:**
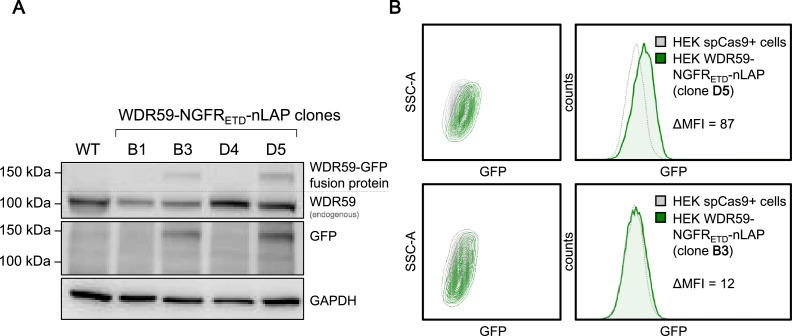
NGFR_ETD_-nLAP-tagged WDR59 expression in HEK spCas9^+^ single cell clones. **A** nLaP-tagged WDR59 protein expression visualized on Western blots probed with anti-WDR59 antibodies and anti-GFP antibodies. GAPDH serves as loading control. **B** Representative FACS profile of selected nLAP-tagged WDR59 expressing HEK WDR59-NGFR_ETD_-nLAP cell clones (D5, upper panel and B3, lower panel). GFP fluorescence (Ex_488nm_) was measured through 530/30 nm bandpass filter. The flow cytometry results were analyzed using FlowJo™ v7.6.5 Software (BD Life Sciences). MFI, mean fluorescence intensity; WT, wild type

In WDR59-NGFR_ETD_-nLAP clone D4 the WT sequence was preserved (PCR B). Two out of four WDR59-NGFR_ETD_-nLAP clones had an insertion of 1 bp (clone B3 and D5) and clone B1 had a deletion of 9 bp. However, as these indels all are located upstream of the initiating ATG, the coding sequence of WT WDR59 is unaffected in all clones, suggesting that these indels do not interfere with WT protein expression (see Fig. 4A). All four WDR59-NGFR_ETD_-nLAP clones exhibited correct tag integrations at the 5’-junction (N-terminus) of the tag (PCR C) (see Table 1).

To verify the expression of tagged WDR59, we subjected PCR-confirmed clones to Western blot analysis with anti-GFP antibodies and anti-WDR59 antibodies (Fig. 4A).

For flow cytometry, nLAP-tagged WDR59 of HEK WDR59-NGFR_ETD_-nLAP cell clones D5 and B3 were selected to measure GFP fluorescence of tagged WDR59. Cell clones B3 and D5 were selected due to higher GFP expression compared to clones B1 or D4. WT HEK293T cells (HEK spCas9^+^) served as negative controls (Fig. 4B).

### Tagged proteins are expressed in HEK spCas9^+^ cells and assembled to complexes

**Fig. 5:**
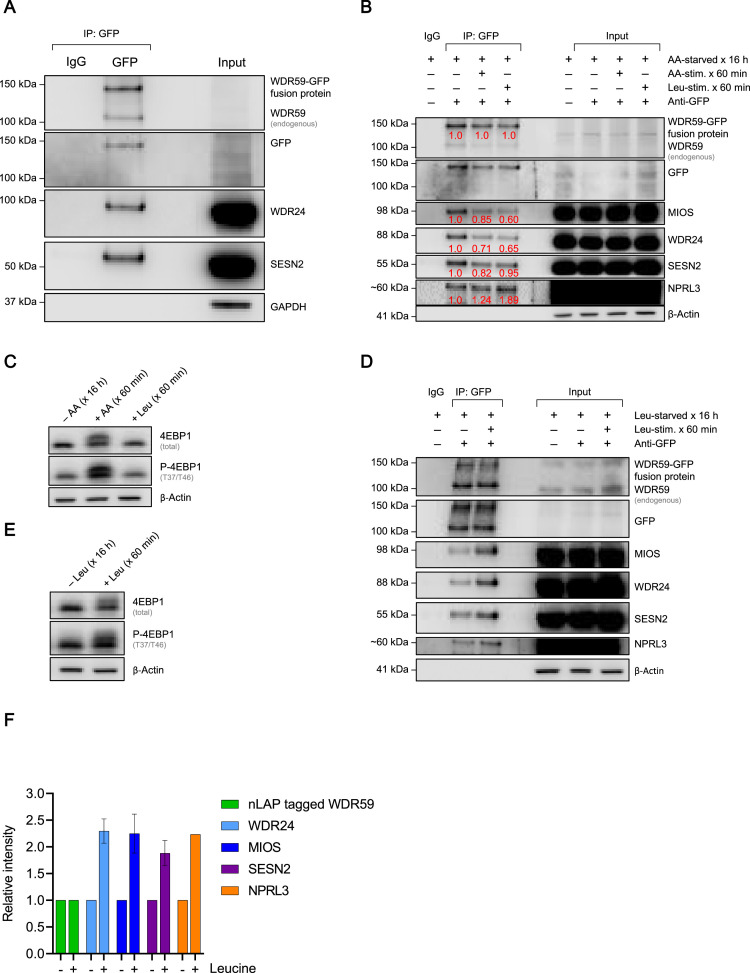
Binding of native GATOR2 interacting proteins to nLAP-tagged WDR59. nLAP-WDR59 was immunoprecipitated from WDR59-NGFR_ETD_-nLAP clone D5 using anti-EGFP antibodies (EGFP-TrapA beads). Endogenous WDR59 and GATOR2 interactors were identified on Western blots with high affinity anti-WDR59, anti-WDR24, anti-MIOS, anti-NPRL3 and anti-SESN2 antibodies, respectively. **A** Known interactors (WDR24, SESN2) were identified by Western bloting using high affinity anti-WDR24 and anti-SESN2 antibodies, respectively. GAPDH was used as control. **B** CoIP of WDR24, MIOS (GATOR2), NPRL3 (GATOR1) as well as SESN2 with nLAP-tagged WDR59 IP in the absence of amino acids and after stimulation with amino acids or leucine for 60 min. Stimulation with all amino acids or leucine decreases interaction between WDR59 (GATOR2) and other GATOR2 subunits (WDR24 or MIOS), as well as between WDR59 and SESN2 as indicated with percentages, normalized to WDR59-GFP signals and to amino acid-starved samples. The interaction with NPRL3 (GATOR1) is increased by stimulation. β-Actin was used as loading control. **C** Amino acid-/ leucine-dependent mTORC1 activity was determined by phosphorylation of 4EPB1 (T37/T46). β-Actin was used as loading control **D** Co-immunoprecipitation (CoIP) of WDR24 and MIOS (GATOR2), NPRL3 (GATOR1) as well as leucine-sensing SESN2 with nLAP-tagged WDR59 IP in the absence of leucine and after stimulation with leucine for 60 min. Leucine stimulation increases binding of WDR59 to WDR24, MIOS, SESN2 and NPRL3 (GATOR1). β-Actin was used as control. **E** Leucine-dependent mTORC1 activity was determined by phosphorylation of 4EPB1 (T37/T46). β-Actin was used as loading control **F** Quantification of CoIP (D), shown is the relative intensity normalized to nLAP-tagged WDR59 signal and to leucine-starved samples; n=2, data presented as mean ± SEM. Abbreviations: DEPDC5, DEP Domain Containing 5; GATOR, GAP activity towards RAG; MIOS, Meiosis Regulator For Oocyte Development (Homolog (Drosophila)); NPRL2/3, Nitrogen Permease Regulator-Like Protein 2/3; SEC13 Homolog, Nuclear Pore And COPII Coat Complex Component; SEH1L, SEH1 Like Nucleoporin; SESN2, Sestrin-2; WDR24/59, WD Repeat Domain 24/59

To show whether nLAP-tagged WDR59 reproduce the protein-protein interactions described for the GATOR2 complex, we selected one clone (nLAP-WDR59 clone D5) for co-immunoprecipitation (CoIP) experiments (Fig. 5). Using GFP specific nanobodies (ChromoTek GFP-Trap®), nLAP-tagged WDR59 (WDR59-GFP) could co-precipitate the GATOR2 subunit WDR24, and is associated with the known GATOR2 interaction partner SESN2 [10] (Fig. 5A). In addition, by enriching the proteins in the IP, a stronger GFP signal was detected as compared to the Western blot analysis of nLAP-tagged WDR59 expression shown in Fig. 4A.

In a next step, endogenously nLAP-tagged WDR59 was used for verification of metabolically-regulated protein-protein interactions. The interaction between GATOR2 (overexpressed WDR24^FLAG^) and SESN2 has been proposed previously as a cellular mechanism to sense the cytosolic level of the amino acid leucine in which leucine has been described to disrupt the SESN2-WDR24^FLAG^ interaction by binding directly to SESN2 [4]. The SESN2-free GATOR2 complex in turn inhibits GATOR1, consisting of Nitrogen Permease Regulator 2 and 3-Like Protein (NPRL2 and 3) and DEP Domain-Containing 5 (DEPCD5), leading to leucine-dependent activation of mTORC1. In times of leucine scarcity, the interaction of SESN2 to GATOR2 hence has to be increased, which probably weakens the GATOR2-mediated inhibition of GATOR1, impeding mTORC1 lysosomal recruitment and activation. To confirm this leucine-regulated SESN2-GATOR2 interaction under physiological conditions, we used the endogenously nLAP-tagged WDR59 to co-immunoprecipitate WDR24 and MIOS (GATOR2), NPRL3 (GATOR1) and SESN2 after full amino acid starvation for 16 hours, followed by 60 minutes of stimulation with all amino acids or leucine only (Fig. 5B). Under amino acid deprived conditions, an interaction of nLAP-tagged WDR59 with SESN2, as well as with NPRL3 (GATOR1) could be shown. In addition, the integrity of GATOR2 (interaction between WDR59 and WDR24 as well as MIOS) was analyzed. Restimulation with all amino acids or leucine alone reduced the interaction of WDR59 with SESN2 to 82% and 95%, whereas the interaction with NPRL3 was enhanced to 1.2- and 1.89-fold, respectively. These observations are in line with previous publications [4,[10], [11], [12]]. Surprisingly, restimulation with all amino acids or leucine resulted in a decrease in the interaction between WDR59 and MIOS (85% and 60%), as well as between WDR59 and WDR24 (71% and 65%). When comparing the effect of amino acid and leucine restimulation, all amino acids reactivated mTORC1 (as measured by phosphorylation of its downstream target eukaryotic Translation Initiation Factor 4E-Binding Protein 1 (4EBP1) at T37/T46), whereas this was not be achieved by the sole stimulation with leucine (Fig. 5C).

Since full amino acid starvation (for 16 hours) serves as a long-term starvation (stress) condition, in which mTORC1 pathway is described to be less sensitive [13], we repeated the interaction studies under less harsh conditions and removed only leucine from the medium followed by leucine stimulation (Fig. 5D). In contrast to leucine restimulation upon full amino acid starvation (Fig. 5B.), leucine stimulation reactivated mTORC1 (Fig. 5E). Surprisingly, under leucine-deprived conditions, the association of WDR59 with SESN2 and NPRL3, as well as with the GATOR2 subunits WDR24 and MIOS were increased approximately by two-fold upon leucine restimulation. The relative intensity of individual interactions with nLAP-tagged WDR59 fusion proteins in response to leucine-signal was quantified and normalized to nLAP-tagged WDR59 IP and leucine-starved samples (Fig. 5F).

Altogether, the results using endogenously nLAP-tagged WDR59 to examine different metabolically controlled protein-protein interactions on the endogenous level strongly indicate, that leucine strengthens the SESN2-GATOR2-GATOR1 assembly in leucine-deprived HEK cells, which is sufficient to reactivate mTORC1 (Fig. 5E). In contrast, restimulation with amino acids or leucine in amino acid-starved cells induced a reduced association of WDR59 with SESN2 as well as with the other GATOR2 subunits WDR24 and MIOS. Furthermore, mTORC1-activity is only restored when all amino acids are available (Fig. 5C).

## Conclusion/Discussion

The advantage of using tagged proteins in protein-protein interaction studies is the ease with which co-immunoprecipitations can be performed using tag-specific high-affinity antibodies. This avoids side effects due to unspecific antibody binding often seen using antibodies directed against the POI. The subsequent protein precipitations allow the detection of interaction partners of the tagged protein with high precision and reflect the natural dynamics within a protein complex and additional interaction partners. Ideally, tagged proteins will reproduce the interactome of their native counterparts, but that is not always necessarily reliable, especially when these fusion proteins are ectopically overexpressed from cDNAs, which is still a widely used strategy. In contrast, endogenous tagging allows physiological expression by introducing the tag into the endogenous locus, which is thus endogenously controlled. Endogenous tagging is preferably achieved by introducing the tag into a selected target gene locus by the NHEJ pathway, as described by Thöne *et al.* (2019) [1] and Lackner *et al.* (2015) [7]. Not only does this circumvent the time-consuming and labor-intensive production of targeting constructs for homologous recombination in cell lines, this method also enables endogenous tagging in a wide variety of cell lines in which the gene repair pathway homology-directed repair (HDR) plays only a very minor role, thus extending the applicability to almost all cell lines and genes using only a set of six generic vectors. In the consecutive experiments, the resulting precise detectability of protein-protein interactions at the endogenous level can yield more reliable results, especially when the interaction is controlled by specific cellular, e.g. metabolic, signals.

Although the selection of cells with successfully tagged proteins via coupled antibiotic resistances is possible, it generates an enormous number of dying cells, which challenges the sub-cultivation of correct clones, especially when using suspension cells which are difficult to separate from dead cells. To facilitate this, fluorescence activated cell sorting (FACS) offers a good alternative when using a GFP-fusion protein. However, this approach can still be difficult when the POI is a weakly expressed endogenous protein. In this case, GFP fluorescence may be barely distinguishable from the background or may be distorted by interfering signals such as autofluorescence. In this study, we extended the selection method following endogenous tagging by coupling the expression of the tagged proteins with the ectopic expression of the extracellular and transmembrane domains of NGFR (NGFR_ETD_) [2]. The direct staining of this surface antigen with appropriate APC-coupled antibodies generates a suitable signal that differs strongly enough from the background and therefore allows a clear differentiation between fusion protein-expressing cells and WT cells (Fig. 3A). It should be mentioned that other fluorophores than APC coupled to anti-NGFR antibodies, as well as magnetic beads coupled to anti-NGFR antibodies can be used, which renders this selection method very adaptable to the respective experimental conditions. Furthermore, tags other than GFP can be used without renouncing precise FACS/MACS selection.

As a proof-of-principle experiment, we attempted to employ this toolkit to endogenously tag the GATOR2 subunit WDR59 in HEK293T cells. We thereby generated four independent cell clones and proved the correct integration of the tag at the molecular level. The validation of WDR59-NGFR_ETD_-nLAP cell clone D5 demonstrated native protein-protein interactions of WDR59 fusion proteins with other GATOR2 subunits (e.g. WDR24 or MIOS), as well as with known interaction partners such as SESN2 or the GATOR1 subunit NPRL3 (Fig. 5A, B and D). The results of the co-immunoprecipitation (CoIP) experiments also indicate that WDR59 is in general a weakly expressed protein, as the signal was weak in the input samples but could be well enriched and visualized in the IP samples. This also explains the observed low GFP expression (Fig. 4A) and GFP fluorescence (Fig. 4B). CoIP experiments with tagged WDR59 further showed that the known interactions between nLAP-tagged WDR59 fusion protein and native counterparts can differ depending on the metabolic signals described (Fig. 5B and D). Stimulation of amino acid-starved cells with all amino acids or leucine alone showed a reduced association of SESN2 with WDR59/GATOR2, but also within the entire GATOR2 complex (based on WDR59, WDR24, and MIOS), which has not been described previously (Fig. 5B). In accordance with previous publications ([4,10–12,14]), the restimulation with all amino acids and leucine alone enhanced the interaction between WDR59 (GATOR2) and NPRL3 (GATOR1), thus supporting the assumption that the inhibition of GATOR1 by GATOR2 is dependent on the interaction strength [10,14]. Correspondingly, mTORC1 could be re-activated by amino acids in amino acid-starved cells, although not by the sole stimulation with leucine (Fig. 5C).

To circumvent the long-term amino acid scarcity, we removed only leucine from the medium and analyzed the leucine-dependent interaction with endogenous nLAP-tagged WDR59 (Fig. 5D). Here, nLAP-tagged WDR59 exhibited an increased association with SESN2 and other GATOR1 and -2 subunits upon stimulation with leucine, indicating that a stronger interaction of SESN2-GATOR2-GATOR1 might be necessary to re-activate mTORC1 upon leucine starvation (Fig. 5E). This is in contrast to the published literature [4,10,11], in which stimulation with leucine resulted in leucine-bound SESN2 to dissociate from the GATOR2 complex. However, the leucine-dependent SESN2-GATOR2 interaction has been elucidated using a WDR24-FLAG overexpression construct to precipitate MIOS and SESN2. In this experimental setting, the WDR24^FLAG^-MIOS interaction remained constant upon leucine restimulation, whereas the interaction between overexpressed WDR24^FLAG^ and SESN2 was fully disrupted [4]. The endogenous interaction between WDR59 and SESN2, however, has not been addressed yet. It cannot be ruled out that SESN2 dissociates from WDR24 rather than from WDR59 upon leucine restimulation, however, our results strongly suggest that the endogenous integrity of the GATOR2 complex, especially in distinct metabolic conditions is more complex than described [12].

In conclusion, our observations after stimulating leucine- and amino acid-deficient cells suggest that certain metabolic signals led to previously contradictory results that need to be further addressed. However, our results demonstrated that nLAP-tagged WDR59 reflects the known interactions with the GATOR2 complex and strongly suggest that endogenously tagged proteins are more sensitive to metabolic signals than overexpressed fusion proteins. We confirmed through protein-protein interaction studies that endogenous LAP-tags are compatible with metabolically-regulated native protein functions and that co-expression of surface markers such as NGFR_ETD_ for selection of correct clones does not negatively interfere with the native interactome of tagged proteins.

## Ethics statements

Not applicable

## Data Availability

No data was used for the research described in the article.
